# How does the EQ-5D-5L perform in asthma patients compared with an asthma-specific quality of life questionnaire?

**DOI:** 10.1186/s12890-020-01205-8

**Published:** 2020-06-13

**Authors:** Boglárka L. Szentes, Konrad Schultz, Dennis Nowak, Michael Schuler, Larissa Schwarzkopf

**Affiliations:** 1 Institute of Health Economics and Health Care Management, Helmholtz Zentrum München (GmbH) – German Research Center for Environmental Health, Comprehensive Pneumology Center Munich (CPC-M), Member of the German Center for Lung Research (DZL), Ingolstädter Landstraße 1, 85764 Neuherberg, Germany; 2 Klinik Bad Reichenhall, Center for Rehabilitation, Pulmonology and Orthopedics, Salzburger Str. 8, 83435 Bad Reichenhall, Germany; 3 LMU University of München, Institute and Outpatient Clinic for Occupational, Social and Environmental Medicine, Ziemssenstraße 1, 80336 Munich, Germany; 4 University of Würzburg, Institute of Clinical Epidemiology and Biometry, Sanderring 2, 97070 Würzburg, Germany; 5 IFT – Institut für Therapieforschung, Leopoldstrasse 175, 80804 Munich, Germany

**Keywords:** EQ-5D-5 L, AQLQ, ACT, Asthma, Health-related quality of life, Responsiveness, Reliability, MID

## Abstract

**Background:**

Asthma patients experience impairments in health-related quality of life (HRQL). Interventions are available to improve HRQL. EQ-5D-5L is a common generic tool used to evaluate health interventions. However, there is debate over whether the use of this measure is adequate in asthma patients.

**Methods:**

We used data from 371 asthma patients participating in a pulmonary rehabilitation (PR) program from the EPRA randomized controlled trial. We used four time points: T0 randomization, T1 start PR, T2 end PR, T3 3 months follow-up. We calculated floor and ceiling effects, intra-class correlation (ICC), Cohen’s d, and regression analysis to measure the sensitivity to changes of EQ-5D-5 L (EQ-5D index and Visual Analog Scale (VAS)) and the disease-specific Asthma Quality of Life Questionnaire (AQLQ). Furthermore, we estimated the minimally important difference (MID). Based on the Asthma Control Test (ACT) scores, we defined three groups: 1. ACT-A (ACT> 19) controlled asthma, 2. ACT-B (14 < ACT≤19) not well-controlled asthma, and 3. ACT-C (ACT≤14) very poorly controlled asthma.

**Results:**

Only the EQ-5D index showed ceiling effects at T2 and T3 (32%). ICC (between T0 and T1) was moderate or good for all measures. Cohen’s d at T2 and T3 was better at differentiating between ACT-A and ACT-B than between ACT-B and ACT-C. The EQ-5D index showed moderate effect sizes (0.63–0.75), while AQLQ showed large effect sizes (0.74–1,48). VAS was responsive to pronounced positive and negative ACT changes in every period, and AQLQ mostly to the positive changes, whereas the EQ-5D index was less responsive. We estimated a MID of 0.08 for the EQ-5D index, 12.3 for VAS, and 0.65 for AQLQ.

**Conclusion:**

All presented HRQL tools had good discriminatory power and good reliability. However, EQ-5D-5 L did not react very sensitively to small changes in asthma control. Therefore, we would suggest using supplementary measures in addition to EQ-5D-5 L to evaluate asthma-specific interventions more comprehensively.

**Trial registration:**

German Clinical Trial Register, DRKS00007740 (date of registration: 05/15/2015), https://www.drks.de/drks_web/navigate.do?navigationId=trial.HTML&TRIAL_ID=DRKS00007740. The registration took place prospectively.

## Background

Asthma is a respiratory disease characterized by chronic inflammation of the airways. Asthma patients experience cough, wheeze, and shortness of breath in varying intensity and frequency [1]. This symptom profile is associated with impairments in health-related quality of life (HRQL) [2–4]. These symptoms can be reduced by adequate drug therapy [1] and through several supplementary management strategies (e.g., patient education [5], respiratory physiotherapy [6], and exercise training [7, 8]), which would increase asthma control and thus presumably HRQL as well.

Two groups of HRQL assessment tools exist, disease-specific and generic ones. Disease-specific assessment tools are developed for specific diseases. They mainly focus on the impact of disease symptoms and the related consequences, but might also cover aspects of disease-associated impairments in social participation or emotional and general wellbeing. They enable comparisons between patients at different stages of the same disease and help to monitor disease development. In contrast, generic assessment tools can be applied across different diseases because they focus on impairments in general health-related aspects of life. Thus, comparisons between different disease areas or with the general population become possible. However, they might not always fully capture HRQL impairments in the context of disease-specific symptoms, especially in the early stages of a disease [9].

One of the most commonly used generic assessment tools is the EQ-5D-5L from the EuroQol group [10], which is a multi-attribute utility instrument (MAUI) for health economic evaluation. It allows the calculation of quality adjusted life years (QALY) [11], an important measure applied in cost–utility studies. Cost–utility studies are approaches, which evaluate and compare health interventions by assessing the costs of an intervention (for example, a pulmonary rehabilitation (PR)) in relation to its health effects. Based on this so-called incremental cost-effectiveness ratio and on additional information, a decision about implementation can be made. Another important aspect to facilitate this decision is the concept of minimally important difference (MID). According to Jaeschke et al. [12], the MID reflects “the smallest difference in score in the domain of interest which patients perceive as beneficial and which would mandate, in the absence of troublesome side effects and excessive cost, a change in the patient’s management.” QALYs and MIDs reflect strategies that take into account different points of view to support decision making in the health care sector, and both approaches have their own reasons for being. Different countries set different priorities regarding the use of one or the other strategy. Furthermore, different stake holders (policy decision makers, clinicians, payers) and different research questions might favor one or the other parameter.

There is debate over whether the use of the generic EQ-5D is adequate in asthma patients. Whalley et al. The three-level version has already raised some concerns, e.g., its inefficient ability to differentiate between different levels of asthma control [13] or that it might miss clinically important changes in asthma control, which is closely associated with higher HRQL [14] . To overcome this issue, a five-level version of the EQ-5D, the EQ-5D-5 L, was developed, which allows more flexibility regarding the description of health states. Thus, a higher sensitivity to change was expected. However, based on a qualitative study in asthma patients, Whalley et al. [15] argued that, even after refinement of the levels, the dimensions per se are lacking in some asthma-relevant aspects. Furthermore, Hyland et al. [16] criticized the low correlation of EQ-5D-5L with lung function values. Hernandez et al. evaluated the metric properties of the EQ-5D-5 L in a cross-sectional setting to confirm the previous results [17]. They found good construct validity and good discriminative ability between health-related groups. Nevertheless, they did not assess responsiveness to changes and did not compare the EQ-5D-5L with a disease-specific assessment tool.

Therefore, our aim is to investigate whether the EQ-5D-5L is suited to measure HRQL in asthma patients in a longitudinal setting, whether it is reliable, and if it is responsive to changes in asthma control, compared with the established disease-specific Asthma Quality of Life Questionnaire (AQLQ). Furthermore, we aim to provide a MID value for the five-level version for asthma patients, which has not to our knowledge been provided in previous studies.

## Methods

**Fig. 1:**
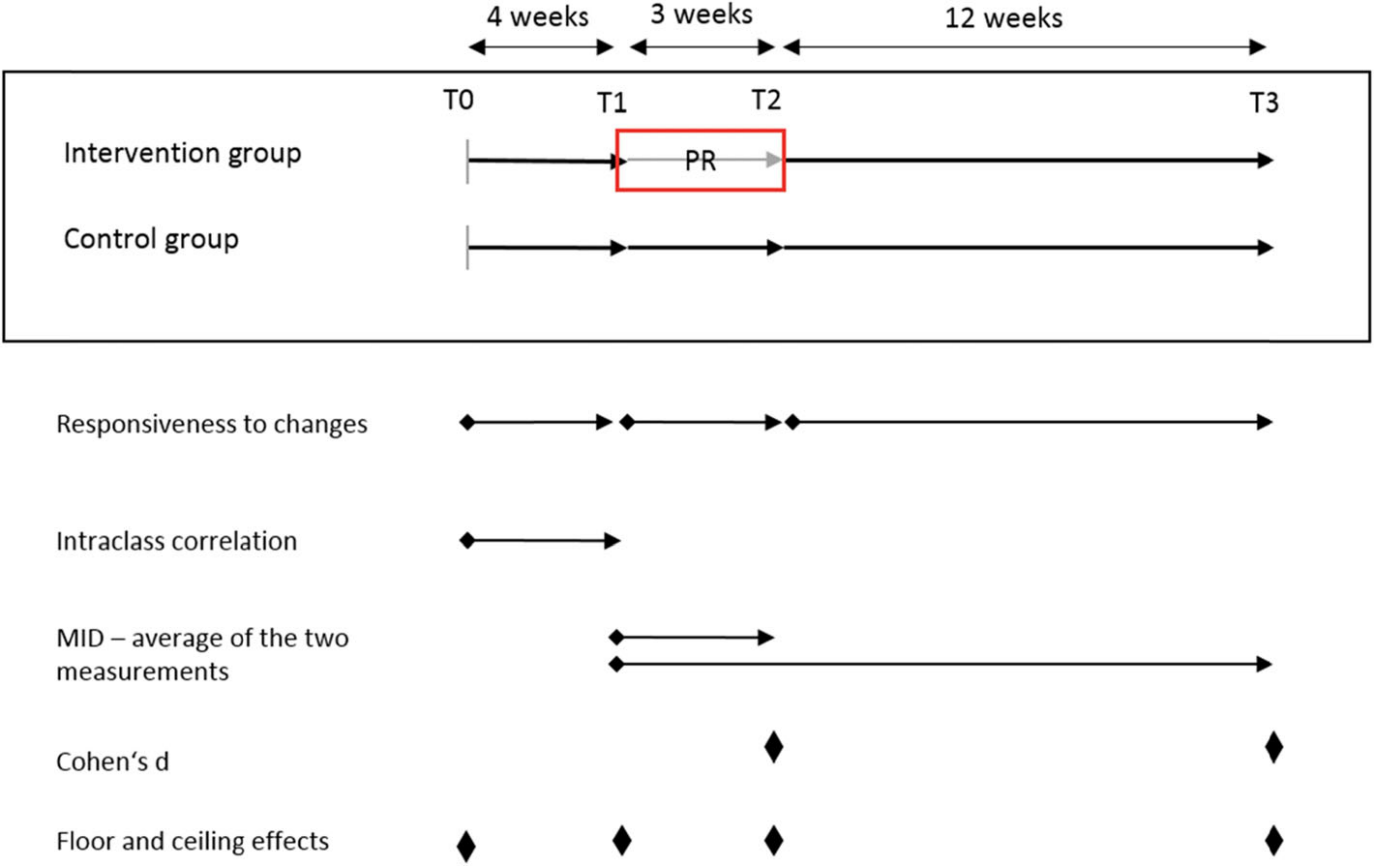
Study design of the RCT and time points of the conducted pooled statistical analyses. Abbreviations: PR: pulmonary rehabilitation, T0: randomization, T1: start PR, T2: end PR, T3: 12 weeks follow-up

We used data from the EPRA study, a randomized controlled trial (RCT) using a wait-list control group assessing the effectiveness of PR among asthma patients (Registered in Deutschen Register Klinischer Studien No. DRKS00007740, the ethics committee of Bayerischen Landesärztekammer approved the study No. 15017). After approval for rehabilitation (T0), patients were randomized to the intervention group (IG) or control group (CG). The IG started the 3-week PR 4 weeks after randomization (T1: start of PR; T2: end of PR), whereas the CG started PR 5 months after randomization (T3). Further details of the study have been published elsewhere [18]. We assessed HRQL and asthma control at T0, T1, T2, and T3 in both groups. For the subsequent analyses, we only included patients with no missing values in the HRQL measures at any time point until T3 to avoid bias through imputation. Furthermore, we pooled the data from both groups. Figure 1 shows the timeline and the time point of the statistical tests described in the statistical analysis section.

We assessed disease severity and HRQL using the following measures:

### Asthma control test (ACT)

The ACT is a self-administered questionnaire to evaluate asthma control [19]. It contains five questions with five possible answers addressing asthma symptoms in the previous 4 weeks. The sum score ranges between 5 and 25; values > 19 represent controlled asthma, and values < 20 are regarded as uncontrolled not well-controlled asthma, as defined by the GINA guidelines [20]. A change of three points is regarded as a MID [21]. For parts of our analyses, we grouped patients into three categories according to their achieved ACT score: ACT-A as well-controlled asthma (ACT score > 19), ACT-B as not well-controlled asthma (16–19), and ACT-C as very poorly controlled asthma (5–15).

### Asthma quality of life questionnaire (AQLQ)

The standardized version of the AQLQ is an asthma-specific HRQL assessment tool containing 32 questions in four domains (symptoms, activity limitations, emotional function, and environmental exposure) [22, 23]. The questions cover the last 2 weeks prior to the survey. Each question has to be answered on a 7-point Likert scale. The overall score ranges between 1 and 7, with the latter indicating the best HRQL. A change of 0.5 points is regarded as a MID [24].

### EQ-5D-5L

The EQ-5D-5L is a generic HRQL measure from the EuroQol group [25], which evaluates the current health state of the patients. It consists of two parts: The first part is the EQ-5D descriptive system with five dimensions (mobility, self-care, usual activities, pain/discomfort, and anxiety/depression); each represented by five different levels (from experiencing no problems to extreme problems). Combining the dimension-specific levels across the five dimensions yields distinct health states, which form the basis for a preference-based valuation (utility). Country-specific tariffs exist for this valuation. We used the German Tariff from Ludwig et al. [26], which ranges between − 0.661 and 1; the higher the value, the better the HRQL. The second part of the EQ-5D-5L is the visual analog scale (VAS). The VAS is a vertical thermometer assessing self-rated health with values from 1 to 100, with 100 indicating the best HRQL.

### Global rating of change scale (GROC)

The GROC is a rating scale with 15 categories assessing the self-reported change in global health. Patients with improvement and deterioration are symmetrically distributed around zero [12, 27], with negative values representing deterioration and positive values representing improvement. We grouped patients according to their perceived changes into four groups following Juniper et al. [24]: “no change” (GROC [− 1; 1], “small change” (GROC [− 3; − 2] and [2; 3]), “moderate change” (GROC [− 5; − 4] and [4; 5]), and “large change” (GROC [− 7; − 6, 6; 7]). Additionally, we split those groups according to the direction of change to calculate a MID for deterioration and for improvement. We assessed the GROC at T2 and T3 (reference to change was the health state at T1 in both cases).

### Statistical analysis and assessing measurement properties

All analyses were performed with SAS (SAS Institute Inc., Cary, NC, USA, version 9.4), and p-values of 0.05 or less were considered statistically significant. We looked at floor and ceiling effects at every time point, defined as > 15% of the patients reaching the best/worst HRQL score [28]. Furthermore, we calculated known-group validity, intra-class correlation (ICC), responsiveness to ACT changes, and the MID.

### Known-group validity

Known-group validity (Cohen’s d) is used to evaluate the ability of the HRQL tools to differentiate between disease severity groups. Cohen’s d was assessed as the mean adjusted differences in HRQL scales between the ACT groups, divided by their pooled standard deviation at T2 or T3. We adjusted for group (IG/CG), age, sex, smoking status, body mass index (BMI), and employment status before PR (yes/no) to compensate for changes not originating from a change in ACT. Cohen’s d was considered small between 0.2 and 0.5, moderate from 0.5 to 0.8, and large above 0.8 [29].

### Intra-class correlation

To estimate the reliability of the HRQL questionnaires, we evaluated ICC (two-way random effects, absolute agreement, single rater) [30] between T0 and T1 for patients who were stable according to their ACT. We considered patients as stable if their ACT score changed by less than the MID. ICC > 0.9 was regarded as high, 0.75–0.9 as good, 0.5–0.75 as moderate, and < 0.5 as poor [31].

### **Responsiveness to ACT change**

To estimate the responsiveness of HRQL scales associated with a change in ACT, we conducted different regression analyses for each HRQL scale. The dependent variable was the HRQL change score (ΔHRQL) in three periods (period 1: T1–T0, period 2: T2–T1, and period 3: T3–T2). The independent variables were ACT change (ΔACT) in five categories (ΔACT ≥MID, 0 < ΔACT<MID, ΔACT = 0, 0 > ΔACT≥MID, ΔACT≤MID) in the respective period, group (IG/CG), age, sex, BMI, smoking status, employed before PR (yes/no), and previous HRQL at T0, T1, or T2 respectively. ∆ACT = 0 was the reference group. The ACT categories are based on the approach of Sullivan et al. [14], who analyzed the responsiveness of the EQ-5D and an asthma-specific questionnaire to changes in asthma control. As a sensitivity analysis, we calculated a quantile regression model for the quantiles 0.5 and for the extremes 0.1 and 0.9, which enables us to portray varying reactions to a continuous ACT change. As there is no hard evidence for the relationship to be linear, considering reactions at different starting points might give deeper insights. This analysis included the same adjustment variables.

### **Minimal important difference (MID)**

We measured the GROC at T2 and T3 and considered a small GROC change as the minimal important change. We calculated the MID separately for improvement and deterioration, as well as combined using the absolute value of the changes. In analogy to Juniper et al. [24], who analyzed MIDs for the AQLQ, the mean of the two measurements (T2 and T3) was considered as the MID. This analysis strategy creates comparability between the disease-specific and generic HRQL tools and enables a cross-validation of our results with existing MIDs for AQLQ.

## Results

### Study population

The study sample included 371 patients: 199 (53.6%) were in the CG and 172 (46.4%) in the IG. The mean age was 51.4 years (SD: 5.6), and 58.5% of the population was male. Around 50% of the patients were current or previous smokers, and more than 80% were employed before the PR. Baseline HRQL did not differ in the groups, HRQL gains of the IG exceeded that of the CG regarding every measure. The whole development of the HRQL stratified by groups can be seen in Table 1, along with further characteristics.

**Table 1:** Characteristics of the study population stratified by group

			**All**	**Control group**	**Intervention group**
**N (%)**	T0		**371**	**199 (53.6)**	**172 (46.4)**
**Male***N (%)*	T0		217 (58.5)	112 (56.3)	105 (61.1)
**Age***Mean (SD)*	T0		51.4 (8.6)	51.4 (8.6)	51.4 (8.6)
**BMI***Mean (SD)*	T0		29.8 (5.9)	30.3 (5.8)	29.1 (6.1)
**Smoking status**	T0	Current smoker	50 (13.5)	26 (13.1)	24 (14.0)
		Ex-smoker	142 (38.3)	69 (34.7)	73 (42.4)
		Never smoker	179 (48.3)	104 (52.3)	75 (43.6)
**Employed (yes)***N (%)*	T0		322 (86.8)	176 (88.4)	146 (84.9)
**ACT**	T0		13.2 (3.7)	13.3 (3.8)	13.1 (3.5)
*Mean (SD)*	T1		15.1 (4.1)	14.8 (4.0)	15.5 (4.1)
	T2		18.1 (5.0)	15.3 (4.6)	21.4 (3.2)
	T3		18.0 (4.8)	15.8 (4.2)	20.6 (4.0)
**AQLQ**	T0		3.97 (0.93)	3.92 (0.91)	4.03 (0.95)
*Mean (SD)*	T1		4.21 (1.00)	4.09 (1.02)	4.36 (0.97)
	T2		4.87 (1.22)	4.19 (1.00)	5.66 (0.96)
	T3		4.90 (1.18)	4.41 (1.02)	5.47 (1.09)
**EQ-5D index**	T0		0.77 (0.20)	0.77 (0.19)	0.77 (0.21)
*Mean (SD)*	T1		0.80 (0.19)	0.78 (0.19)	0.82 (0.19)
	T2		0.84 (0.18)	0.79 (0.20)	0.90 (0.15)
	T3		0.84 (0.20)	0.80 (0.20)	0.88 (0.18)
**VAS**	T0		57.2 (16.9)	57.0 (17.6)	57.5 (16.2)
*Mean (SD)*	T1		60.3 (17.4)	59.6 (18.5)	61.2 (16.2)
	T2		68.0 (19.4)	58.6 (18.5)	78.9 (14.1)
	T3		67.1 (19.1)	59.2 (17.6)	76.2 (16.6)

Abbreviations: *BMI* body mass index, *ACT* Asthma Control Test, *AQLQ* Asthma Quality of Life Questionnaire, *VAS* Visual Analog Scale

### Properties of the HRQL questionnaires

#### Floor and ceiling effects

None of the questionnaires used showed floor effects at any time point. Only the EQ-5D index showed ceiling effects at T2 and T3 with 55 (32%) patients each (Additional file 1).

#### Reliability

AQLQ and the EQ-5D index showed a good ICC (0.82, 95% confidence interval (CI) [0.78; 0.886] and 0.78 CI [0.72; 0.83]); VAS showed moderate ICC (0.62 CI [0.53: 0.70]).

#### Known-group validity

**Fig. 2:**
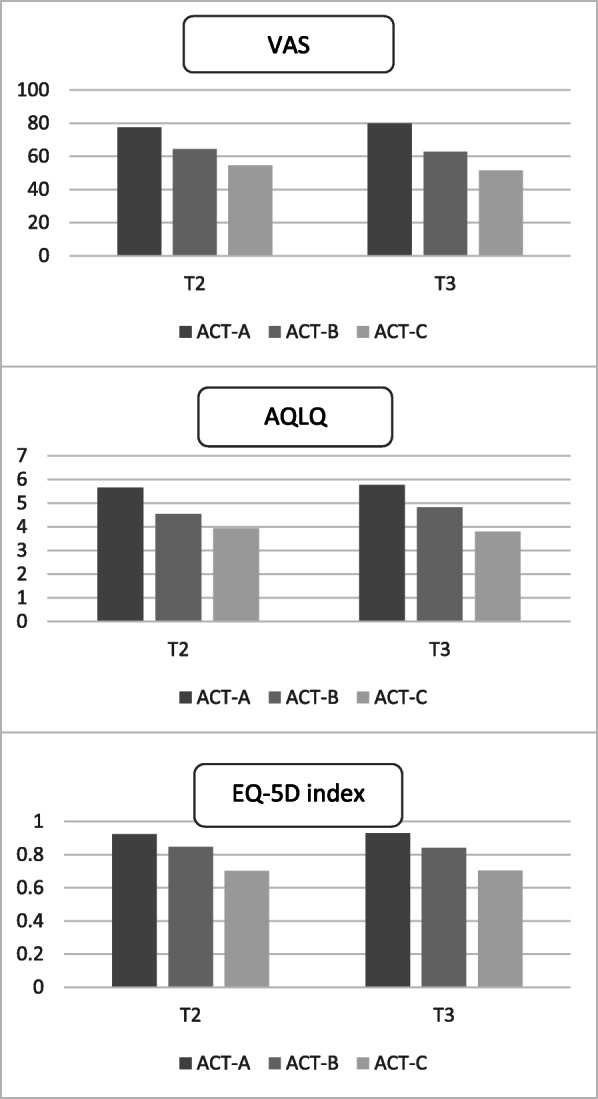
Adjusted mean scores for the ACT groups at T2 and T3. All differences between the groups were significant at the 0.05 level. Abbreviations: ACT: Asthma Control Test, ACT-A: well-controlled asthma (ACT score > 19), ACT-B: not well-controlled asthma (16–19), and ACT-C: very poorly controlled asthma (5–15)

At T2, there were 185 (49.9%) patients in ACT-A, 72 (19.4%) in ACT-B, and 114 (30.7%) in ACT-C. At T3, there were 164 (44.2%) patients in ACT-A, 94 (25.3%) in ACT-B, and 113 (30.5%) in ACT-C. Adjusted mean scores for the ACT groups at T2 and T3 can be found in Fig. 2. Cohen’s d was similar for the EQ-5D index at every measuring point, whereas VAS was able to discriminate better between well-controlled asthma and not well-controlled asthma than between more severe cases. A similar pattern emerged for AQLQ, but with mostly higher values. Further details on Cohen’s d are presented in Table 2.

**Table 2:** Known-group validity at T2 and T3

**Cohen’s d**		**ACT A vs. ACT-B**	**ACT B vs. ACT-C**
**T2**	AQLQ	1.48	0.74
	EQ-5D index	0.72	0.73
	VAS	0.97	0.57
**T3**	AQLQ	1.25	1.35
	EQ-5D index	0.75	0.63
	VAS	1.33	0.70

Abbreviations: *AQLQ* Asthma Quality of Life Questionnaire, *VAS* Visual Analog Scale, *ACT* Asthma Control Test, *ACT-A* well-controlled asthma (ACT score > 19), *ACT-B* not well-controlled asthma (16–19), and *ACT-C* very poorly controlled asthma (5–15)

#### Responsiveness

The overall responsiveness of a change in asthma control (measured in categories) of the HRQL tools was moderate. In most cases, AQLQ and VAS could differentiate between patients staying stable vs. patients reaching the |MID| on the ACT scale. The EQ-5D index was responsive to changes in only one period (period 3, detecting high negative changes) (Table 3). However, the confidence intervals between adjacent groups frequently overlapped, providing less reliable results for all HRQL measures (Table 3). The sensitivity analysis showed that every HRQL tool reacts positively to an increase in ACT (Table 4); however, the EQ-5D index and AQLQ were not significant in quantile 0.1. Furthermore, there was a gradient change of HRQL in AQLQ and the EQ-5D index through the quantiles, but VAS turned out to be more volatile.

**Table 3:** Responsiveness of the different HRQL measures to changes in ACT—results of the regression analyses

		**N (%)**	**β-coefficient**	***p*-value**	**95% confidence intervals**	
**AQLQ change**	3 ≤ ∆ACT	185 (49.9)	**0.63**	< 0.0001	0.417	0.850
**(T2–T1)**	0 < ∆ACT< 3	*66 (17.8)*	**0.03**	0.824	−0.199	0.250
	*∆ACT = 0*	*41 (11.1)*	**Ref. cat.**	*.*	*.*	*.*
	−3 < ∆ACT< 0	*50 (13.5)*	**−0.14**	0.226	−0.374	0.089
	∆ACT≤–3	*29 (7.8)*	**−0.23**	0.095	−0.494	0.040
**AQLQ change**	3 ≤ ∆ACT	*66 (17.8)*	**0.25**	0.021	0.037	0.464
**(T3–T2)**	0 < ∆ACT< 3	*87 (23.5)*	**0.03**	0.776	−0.171	0.229
	*∆ACT = 0*	*54 (14.6)*	**Ref. cat.**	.	.	.
	−3 < ∆ACT< 0	*93 (25.1)*	**−0.34**	0.0006	−0.532	− 0.145
	∆ACT≤–3	*71 (19.1)*	**−0.84**	< 0.0001	−1.047	−0.632
**EQ-5D index change**	3 ≤ ∆ACT	*185 (49.9)*	**0.04**	0.089	−0.006	0.090
**(T2–T1)**	0 < ∆ACT< 3	*66 (17.8)*	**0.01**	0.663	−0.039	0.061
	*∆ACT = 0*	*41 (11.1)*	**Ref. cat.**	*.*	*.*	*.*
	−3 < ∆ACT< 0	*50 (13.5)*	**0.01**	0.637	−0.039	0.064
	∆ACT≤–3	*29 (7.8)*	**−0.02**	0.419	−0.084	0.035
**EQ-5D index change**	3 ≤ ∆ACT	*66 (17.8)*	**0.02**	0.295	−0.022	0.071
**(T3–T2)**	0 < ∆ACT< 3	*87 (23.5)*	**−0.01**	0.691	−0.052	0.034
	*∆ACT = 0*	*54 (14.6)*	**Ref. cat**	.	.	.
	−3 < ∆ACT< 0	*93 (25.1)*	**−0.02**	0.398	− 0.060	0.024
	∆ACT≤–3	*71 (19.1)*	**−0.08**	0.0008	−0.123	−0.033
**VAS change**	3 ≤ ∆ACT	*185 (49.9)*	**5.82**	0.024	0.782	10.850
**(T2–T1)**	0 < ∆ACT< 3	*66 (17.8)*	**−1.62**	0.540	−6.809	3.572
	*∆ACT = 0*	*41 (11.1)*	**Ref. cat.**	***.***	***.***	***.***
	−3 < ∆ACT< 0	*50 (13.5)*	**−5.29**	0.054	−10.658	0.084
	∆ACT≤–3	*29 (7.8)*	**−7.77**	0.014	−13.946	−1.592
**VAS change**	3 ≤ ∆ACT	*66 (17.8)*	**5.96**	0.009	1.507	10.423
**(T3–T2)**	0 < ∆ACT< 3	*87 (23.5)*	**2.26**	0.286	−1.900	6.424
	*∆ACT = 0*	*54 (14.6)*	**Ref. cat.**	.	.	.
	−3 < ∆ACT< 0	*93 (25.1)*	**−0.69**	0.736	−4.726	3.340
	∆ACT≤–3	*71 (19.1)*	**−8.97**	< 0.0001	−13.298	−4.650

All results are adjusted for group (intervention vs. control), age, sex, smoking status, BMI, employed (yes/no), and HRQL at T0, T1, or T2 respectively. MID for ACT = 3. Abbreviations: *ACT* Asthma Control Test, *AQLQ* Asthma Quality of Life Questionnaire, *Ref. cat*. reference category, *VAS* Visual Analog Scale

**Table 4:** Responsiveness of the HRQL measures to continuous changes in ACT

		**T1-T0**			**T2-T1**			**T3-T2**		
		**Estimate**	**Stand. error**	***p*-value**	**Estimate**	**Stand. error**	***p*-value**	**Estimate**	**Stand. error**	***p*-value**
**AQLQ**	Q0.1	0.03	0.01	0.125	0.08	0.02	< 0.0001	0.13	0.01	< 0.0001
	Q0.5	0.05	0.01	< 0.0001	0.09	0.01	< 0.0001	0.10	0.01	< 0.0001
	Q0.9	0.05	0.02	0.003	0.09	0.02	< 0.0001	0.01	0.02	< 0.0001
**EQ-5D index**	Q0.1	0.003	0.004	0.414	0.009	0.002	< 0.0001	0.014	0.004	0.0002
	Q0.5	0.005	0.001	0.0005	0.005	0.001	0.0001	0.009	0.002	< 0.0001
	Q0.9	0.005	0.002	0.03	0.0014	0.001	0.171	0.004	0.001	0.008
**VAS**	Q0.1	1.12	0.40	0.005	1.65	0.31	< 0.0001	1.74	0.35	< 0.0001
	Q0.5	1.00	0.24	< 0.0001	1.1	0.23	< 0.0001	1.37	0.25	< 0.0001
	Q0.9	1.08	0.34	0.002	0.68	0.24	0.006	1.13	0.27	< 0.0001

All results are adjusted for group (intervention vs. control), age, sex, smoking status, BMI, employed (yes/no), and HRQL at T0, T1, or T2 respectively. Abbreviations: Stand. error: standard error, ACT: Asthma Control Test, AQLQ: Asthma Quality of Life Questionnaire, VAS: Visual Analog Scale

#### MID

According to GROC at two time points, we identified (combining deterioration and improvement) mean MIDs in the pooled analysis of 0.67 [0.61; 0.74] for AQLQ, 12.28 [10.94; 13.61] for VAS, and 0.09 [0.07; 0.1] for the EQ-5D index (Table 5). Except for the EQ-5D index, we examined a gradient change in HRQL with increasing magnitude of the GROC change. In the analyses stratified for direction of change, the gradient changes appeared in all HRQL measures with regard to improvement. In case of deterioration, a large negative change was associated with positive values in the first measurement, except for the VAS. At the second measurement (T1–T3), the gradient change was detectable for every tool for deterioration and improvement.

**Table 5:** Mean change in HRQL scores stratified by GROC

**T1–T2** ***N*** **= 371**	**Distinction between deterioration and improvement**							**No distinction**			
***N*** **= 371**	**Large neg** ***n*** **= 6**	**Mod. neg*n*** **= 13**	**Small neg** ***n*** **= 43**	**No change*n*** **= 107**	**Small pos*n*** **= 52**	**Mod. pos** ***n*** **= 96**	**Large pos** ***n*** **= 54**	**No change** ***n*** **= 107**	**Small change** ***n*** **= 95**	**Mod. change** ***n*** **= 109**	**Large change** ***n*** **= 60**
**AQLQ change*Mean [CI]***	0.02 [− 0.25; 0.29]	−0.35 [− 0.69; − 0.01]	−0.04 [− 0.2; 0.12]	0.14 [0.05; 0.24]	0.62 [0.40; 0.83]	1.13 [0.99; 1.27]	1.78 [1.58; 1.97]	0.38 [0.32; 0.44]	0.60 [0.49; 0.71]	1.05 [0.92; 1.19]	1.62 [1.4; 1.83]
**EQ-5D index change*Mean [CI]***	0.04 [−0.35; 0.44]	−0.07 [− 0.17; 0.03]	−0.02 [− 0.06; 0.02]	0.00 [− 0.02; 0.03]	0.04 [0.00; 0.08]	0.08 [0.06; 0.11]	0.08 [0.05; 0.11]	0.08 [0.06; 0.1]	0.08 [0.06;0.11]	0.11 [0.09;0.13]	0.11 [0.08; 0.14]
**VAS change*Mean [CI]***	−6.67 [−16.98; 3.65]	−9.62 [−22.24; 3.01]	−7.14 [− 11.72; − 2.56]	− 0.45 [−3.22; 2.32]	9.54 [5.96; 13.12]	17.52 [14.37;20.67]	21.98 [18.10;25.87]	9.42 [7.32; 11.51]	12.2 [10.07;14.37]	18.4 [15.7; 21.12]	20.6 [16.92;24.31]
**T1–T3** ***N*** **= 371**	**Distinction between deterioration and improvement**							**No distinction**			
	**Large neg** ***n*** **= 3**	**Mod. neg*n*** **= 18**	**Small neg*n*** **= 40**	**No change*n*** **= 119**	**Small pos*n*** **= 63**	**Mod. pos** ***n*** **= 85**	**Large pos*n*** **= 43**	**No change** ***n*** **= 119**	**Small change** ***n*** **= 103**	**Mod. change** ***n*** **= 103**	**Large change** ***n*** **= 46**
**AQLQ change*Mean [CI]***	−0.10 [−1.0; 0.77]	−0.03 [− 0.35; 0.29]	0.01 [− 0.17; 0.19]	0.34 [0.23; 0.45]	0.73 [0.57; 0.90]	1.17 [1.0; 1.35]	1.62 [1.34; 1.91]	0.53 [0.45; 0.61]	0.69 [0.6; 0.79]	1.14 [1.02; 1.27]	1.57 [1.3; 1.84]
**EQ-5D index change** ***Mean [CI]***	−0.18 [−1.11; 0.76]	− 0.09 [− 0.22; 0.03]	0.00 [− 0.04; 0.04]	0.02 [− 0.01; 0.04]	0.04 [0.02; 0.07]	0.06 [0.03; 0.09]	0.10 [0.03; 0.16]	0.09 [0.07; 0.11]	0.08 [0.07; 0.1]	0.12 [0.09; 0.14]	0.15 [0.09; 0.2]
**VAS change*Mean [CI]***	−23.33 [−37.68; −8.99]	−7.56 [−16.59; 1.48]	−4.88 [− 10.85; 1.10]	0.61 [− 2.16; 3.39]	7.40 [3.95; 10.85]	17.07 [13.63;20.51]	21.60 [17.12;26.1]	9.6 [7.49; 11.79]	12.3 [10.0; 14.6]	18.1 [15.47;20.79]	22.4 [18.49;26.25]

Abbreviations: *HRQL* health-related quality of life, *GROC* Global Rating of Change, *ACT* Asthma Control Test, *AQLQ* Asthma Quality of Life Questionnaire, *VAS* Visual Analog Scale, *CI* 95% confidence interval, *mod* moderate, *neg* negative, *pos* positive

## Discussion

Our study contributed to the discussion about the suitability of EQ-5D-5 L in measuring asthma severity and asthma development over time. We assessed its reliability, its ability to differentiate between disease severity, and its responsiveness to changes. As a comparator, we used an established disease-specific questionnaire, the AQLQ. Furthermore, we calculated estimates for the MIDs to facilitate the evaluation of interventions in the disease area asthma.

In a cross-sectional setting, AQLQ showed the best discriminatory power between the asthma severity states, although it showed variation across time points. In contrast, Cohen’s d for the EQ-5D index was stable across time points (T2 vs. T3) and different severity levels (ACT-A|ACT-B vs. ACT-B|ACT-C), but lower. Furthermore, AQLQ and VAS had a higher ability to differentiate between patients with asthma control or notand without asthma control (ACT-A vs. ACT-B) compared with differentiating between not well-controlled and very poorly controlled asthma (ACT-B vs. ACT-C). As the goal is to reach asthma control for most of the interventions, the differentiation between different degrees of uncontrolled not controlled asthma might be considered of secondary value. The results suggest that AQLQ, the EQ-5D index as well as VAS are all suited to detect patient groups with low HRQL and greater need for disease control, e.g., patients eligible for PR. Hernandez et al. [17] conducted similar analyses in their study, although using different distinguishing factors, e.g., the number of chronic conditions, asthma control and inhaler use [17]. This makes a comparison of the results difficult. When comparing groups with different asthma control, Hernandez et al. found a better ability of the EQ-5D index to differentiate between the groups compared with VAS [17], which we cannot confirm. Furthermore, the ceiling effect shown in their work is smaller than that we observed (26.5% vs. 32% for the EQ-5D index). The study samples differed in age, female/male ratio, disease severity, and the tariffs used [3, 17]. Additionally, our study sample also included patients with a lower level of asthma control. This might explain the slightly different results.

An important aspect in health economics is the evaluation of health interventions. Therefore, HRQL tools should be reliable and responsive to changes to enable evidence-based recommendations regarding health care interventions. In a longitudinal approach, we assessed reliability (ICC) between T0 and T1, where none of the patients had yet received PR and their ACT score stayed stable. Reliability was moderate for VAS but good for the EQ-5D index and AQLQ. Without interventionA, asthma-related components of HRQL without intervention tend to be more stable than generic health, which might explain the observed higher reliability of the AQLQ. Additionally, AQLQ reflects a time period of 4 weeks, whereas EQ-5D-5 L asks for current health only, which increases the volatility of the measurements. Nevertheless, all instruments are suitable for repeated measurements.

We assume that PR improves asthma control and clinical parameters and thus positively affects (at least disease-specific) HRQL. Therefore, in our pooled analysis, we had subgroups experiencing improvement (mostly in the IG) and patient groups staying relatively stable (mostly in the CG). This allowed us to examine HRQL changes in a heterogeneous study population. AQLQ was sensitive to big positive and negative changes (changes ≥|MID|). VAS was also able to differentiate between patients with deteriorating or improving HRQL by more than the MID-ACT, but not between small negative or positive changes. Given that the reference group for all HRQL tools is “no change”, a detection of changes below MID is very challenging because of the slight differences from the reference level. The EQ-5D index in our sample could not differentiate significantly between patients reaching a clinically relevant change on ACT (MID) or not, except for one case. This might be an issue regarding cost–utility studies using QALYs as the primary outcome, as suggested by the National Institute for Health and Care Excellence guidelines because, even if patients reach a clinically relevant increase in ACT (MID) through an intervention, it might be overlooked by the EQ-5D index. Thus, the intervention would not be considered cost effective. Looking at the quantile regression approach, a slightly different pattern emerged, where the EQ-5D index detects changes. However, we believe that the magnitude of the change on the EQ-5D index does not match the change on the ACT (e.g. at quantile 0.5 a MID change on ACT only changes the EQ-5D index by approximately 20% of its estimated MID), and leaves a significant improvement on the ACT undetected. Cost–utility studies should thus consider other secondary outcomes, which can potentially evaluate these changes. Similar results were reported from Sullivan et al. [14]; however, the comparison is hindered to some extent, as Sullivan et al. used the previous 3L version of EQ-5D. Therefore, a direct comparison is difficult. VAS and the AQLQ could be used to complement the EQ-5D index, as they showed better (although not perfect) responsiveness to changes. However, AQLQ and VAS are not appropriate measures for cost–utility analysis, but for cost-effectiveness analyses only. In our sensitivity analysis, we confirmed that all measurements react positively to an improvement in ACT. Nevertheless, we think that regarding the magnitude of change, teh EQ-5D index does not react sufficiently sensitive to detect important changes in asthma control. Indeed observed changes in EQ. 5D are rather small and might hence mask the parallel substantial changes in ACT.

Using the GROC to identify the MID for the AQLQ resulted in a slightly higher MID than previous literature would suggest (0.65 vs. 0.5) [24]. However, MID calculations usually differ depending on the study population and the calculation method used. As expected, in the case of deterioration, a smaller change is considered clinically relevant than in the case of improvement. This suggests the existence of different MIDs depending on the direction of change. However, the consideration of different MIDs might not be manageable in a clinical setting. Thus, for most indications, a single MID is used. In the combined analysis, the EQ-5D index characterized no change and minimal change with similar values. Consequently, we can assume that the EQ-5D-5L is less suitable to detect changes in the HRQL of patients, as the previous calculations show. Probably, the dimensions are covering life aspects broadly, but they might miss other important aspects related to asthma. To overcome this issue, Whalley et al. suggest, for example, the addition of a respiratory domain to the EQ-5D [15]. Nevertheless, the calculated value (0.08) was close to the simulation-based values from McClure et al. (0.07) [32]. This suggests the validity of our results; however, the low responsiveness to changes in the utilities should be kept in mind. Furthermore, there is an ongoing debate about the use of MID in economic evaluations, because of its narrow definition [33]. Additionally, there are also concerns about the methodological challenges to incorporate HRQL into RCTs (e.g., HRQL tools being preference based), which also have to be kept in mind during interpretation [34]. These results contribute to the controversy described in the introduction about the use of the EQ-5D in asthma patients. Our study cannot comment on the content validity of the EQ-5D, but we can agree that there might be a need to reconsider the five dimensions in this setting, although further research is necessary on this topic. Another possible solution might be the use of a bolt-on method, which amends the EQ-5D with information on the initially missing dimension [35]. However, there is no scientific consensus about the most suitable bolt-on method yet.

Szende et al. [36] used the previous 3L version and showed evidence of ceiling effects [36]. This implies that the discriminative properties of the EQ-5D in patients experiencing good health may not be sufficient. McTaggart-Cowan et al. are addressing similar aspects, questioning the ability of the EQ-5D to discriminate across different disease severity [13]. Although we experienced similar issues, the use of the 5 L version seemed to lower the magnitude of these.

Although the EQ-5D index showed slightly worse properties than the AQLQ, we should be aware of the different approaches behind the questionnaires. Generic questionnaires cover broad life aspects and facilitate comparisons among different disease groups, whereas disease-specific measures are for within-group comparisons. Furthermore, regarding the responsiveness of the tools to an ACT change is easier for the AQLQ, as it measures similar aspects and thus has overlapping content, whereas the EQ-5D index lacks asthma-specific content and can only indirectly measure such a construct [37, 38].

There are some limitations to this study. As the EQ-5D assesses current health, whereas the AQLQ has a timeframe of 2 weeks and the ACT of 4 weeks, there is a potential bias while comparing these measures directly. Because asthma has a varying intensity, depending on the asthma attacks, valuing health on a single day may lead to distorted results.

Additionally, there is a chance that HRQL tools behave differently in the control vs intervention CG vs. the IG, and a stratified analysis would be recommended. To achieve a sufficiently high n, we conducted a pooled analysis, but we think that our adjustment for the group variable best possibly accounted for this issue.

The generalizability of the results is not necessarily given for patients outside Germany. Furthermore, patients with initially controlled asthma were not included in this analysis; therefore, we might miss important aspects about mild asthma cases. Nevertheless, the number of patients in this randomized controlled setting was high, and we believe our results are still valuable for the examined disease group.

## Conclusion

In conclusion, all presented HRQL tools had good discriminatory power and good reliability. However, EQ-5D-5L had difficulties in detecting (particularly small) changes in disease control. Nevertheless, EQ-5D is still an important tool to compare HRQL across disease areas and to facilitate health economic evaluations, also in the field of asthma. Therefore to draw a more comprehensive picture, we would suggest using supplementary measures (e.g., AQLQ) to EQ-5D-5L to evaluate asthma-specific interventions. Nevertheless, it is still an important tool to compare HRQL across disease areas and to facilitate health economic evaluations.

## Supplementary information

**Additional file 1.**

## Data Availability

The datasets generated and analyzed during the current study are not publicly available because they containing information that could compromise research participant privacy, but are available from the corresponding author on reasonable request.

## Abbreviations

ACT: Asthma Control Test
AQLQ: Asthma Quality of Life Questionnaire
CG: Control group
GROC: Global Ratings of Change
HRQL: Health-related quality of life
ICC: Intra-class correlation
IG: Intervention group
MID: Minimal important difference
PR: Pulmonary rehabilitation
QALY: Quality adjusted life years
VAS: Visual Analog Scale
