# Supplementary material for: How does the EQ-5D-5L perform in asthma patients compared with an asthma-specific quality of life questionnaire?
Source: BMC Pulm Med. 2020 Jun 13;20:168. doi: 10.1186/s12890-020-01205-8 (PMC7293124; doi:10.1186/s12890-020-01205-8)
Supplement: Supplementary file 1 — Additional file 1. [file 12890_2020_1205_MOESM1_ESM.docx]

**Supplementary material**

**Additional files**

**Additional file 1: Floor and ceiling effects**

|  | **Control group** | | | | **Intervention group** | | | |
| --- | --- | --- | --- | --- | --- | --- | --- | --- |
|  | **T0** | **T1** | **T2** | **T3** | **T0** | **T1** | **T2** | **T3** |
| AQLQ max n (%) | 0 | 0 | 1 (0.5) | 0 | 0 | 0 | 0 | 2 (1.2) |
| AQLQ min n (%) | 0 | 0 | 0 | 0 | 0 | 0 | 0 | 0 |
| EQ-5D index max n (%) | 7 (3.5) | 8 (4.0) | 11 (5.5) | 12 (6.0) | 10 (5.8) | 18 (10.5) | 55 (32.0) | 55 (32.0) |
| EQ-5D index min n (%) | 0 | 0 | 0 | 0 | 0 | 0 | 0 | 0 |
| VAS max n (%) | 0 | 1 (0.5) | 1 (0.5) | 2 (1.0) | 0 | 2 (1.2) | 2 (1.2) | 3 (1.7) |
| VAS min n (%) | 0 | 0 | 0 | 0 | 0 | 0 | 0 | 0 |

Abbreviations: AQLQ: Asthma Quality of Life Questionnaire, VAS: Visual Analog Scale
